# Detection of virus-specific T cells via ELISpot corroborates early diagnosis in human Borna disease virus 1 (BoDV-1) encephalitis

**DOI:** 10.1007/s15010-024-02246-5

**Published:** 2024-04-12

**Authors:** Markus Bauswein, Ehab Eid, Lisa Eidenschink, Barbara Schmidt, André Gessner, Dennis Tappe, Dániel Cadar, Merle M. Böhmer, Laura Jockel, Nora van Wickeren, Tamara Garibashvili, Isabel Wiesinger, Christina Wendl, Josef G. Heckmann, Klemens Angstwurm, Martin Freyer

**Affiliations:** 1 Institute of Clinical Microbiology and Hygiene, University Hospital Regensburg, Regensburg, Germany; 2 Department of Neurology, University of Regensburg, Bezirksklinikum, Regensburg, Germany; 3 Bernhard Nocht Institute for Tropical Medicine, Hamburg, Germany; 4 German Consiliary Laboratory for Bornaviruses, Bernhard Nocht Institute for Tropical Medicine, Hamburg, Germany; 5 Bavarian Health and Food Safety Authority, Munich, Germany; 6 Institute of Social Medicine and Health Systems Research, Otto-von-Guericke-University, Magdeburg, Germany; 7 Department of Neurology, Klinikum Landshut, Landshut, Germany; 8 Institute of Neuroradiology, University of Regensburg, Bezirksklinikum, Regensburg, Germany

**Keywords:** Borna disease virus 1 (BoDV-1), Encephalitis, Zoonosis, ELISpot, Indirect immune fluorescence assay (iIFA)

## Abstract

**Background:**

Within endemic regions in southern and eastern Germany, Borna disease virus 1 (BoDV-1) causes rare zoonotic spill-over infections in humans, leading to encephalitis with a high case-fatality risk. So far, *intra-vitam* diagnosis has mainly been based on RT-qPCR from cerebrospinal fluid (CSF) and serology, both being associated with diagnostic challenges. Whilst low RNA copy numbers in CSF limit the sensitivity of RT-qPCR from this material, seroconversion often occurs late during the course of the disease.

**Case presentation:**

Here, we report the new case of a 40 − 50 year-old patient in whom the detection of virus-specific T cells via ELISpot corroborated the diagnosis of BoDV-1 infection. The patient showed a typical course of the disease with prodromal symptoms like fever and headaches 2.5 weeks prior to hospital admission, required mechanical ventilation from day three after hospitalisation and remained in deep coma until death ten days after admission.

**Results:**

Infection was first detected by positive RT-qPCR from a CSF sample drawn four days after admission (viral load 890 copies/mL). A positive ELISpot result was obtained from peripheral blood collected on day seven, when virus-specific IgG antibodies were not detectable in serum, possibly due to previous immune adsorption for suspected autoimmune-mediated encephalitis.

**Conclusion:**

This case demonstrates that BoDV-1 ELISpot serves as additional diagnostic tool even in the first week after hospitalisation of patients with BoDV-1 encephalitis.

## Introduction

Since the zoonotic potential of Borna disease virus 1 (BoDV-1) was proven in 2018 [1, 2], approximately 50 human cases within endemic regions in southern and eastern Germany have been reported to German health authorities [3]. Infections lead to severe encephalitis with an extremely high case-fatality risk (> 90%). As reservoir, the bicolored white-toothed shrew (*Crocidura leucodon*) has been identified which sheds the virus via body fluids and excretions [4]. The exact route of transmission to humans is incompletely understood [5]. A retrospective case–control study identified rural residence close to nature in a stand-alone location or on the fringe of a settlement within virus-endemic regions as the most important risk factor [3]. The incubation time is unknown and is estimated to range between several weeks and months [1, 6]. After initial unspecific symptoms like fever and headaches, severe neurological symptoms, such as memory loss, seizures, ataxia, paralysis and deep coma, occur. Changes in cerebrospinal fluid (CSF) parameters include lymphocytic pleocytosis, elevated CSF protein and increased lactate concentrations; normal white blood cell count (WBC) in CSF, however, does not rule out a BoDV-1 infection [7]. In addition, characteristic patterns in cerebral magnetic resonance imaging (cMRI) have been described, especially T2 hyper-intensities within the head of the caudate nucleus and the insula as well as a spread to the limbic system and cortical areas [8]. An unremarkable cMRI, however, does not exclude the diagnosis either, especially at early stages [1, 8, 9]. According to a recently published case definition, the detection of BoDV-1 RNA or protein is required for a confirmed case, whilst the detection of bornavirus-reactive IgG in addition to clinical neurological symptoms is classified as probable case [10]. As the immuno-pathogenesis of the disease is assumed to be driven by virus-specific T cells [11], we recently established a BoDV-1 ELISpot as promising complementary diagnostic test [12]. Here, we describe a newly diagnosed case of human BoDV-1 encephalitis in which a positive BoDV-1 ELISpot corroborated the diagnosis, three days before bornavirus-reactive IgG was detected via an indirect immunofluorescence assay (iIFA).

## Methods

### BoDV-1 RT-qPCR

BoDV-1 RT-qPCR was performed according to a previously published protocol [1]. Total RNA was extracted using the EZ1 Virus Mini Kit v2.0 (Qiagen, Hilden, Germany) on an EZ1 Advanced XL system (Qiagen), according to the manufacturer's instructions. 5 µL eluate was used for one-step reverse transcription and amplification in a total reaction volume of 30 µL using the TaqPath™ 1-Step RT-qPCR Master Mix, CG (Thermo Fisher Scientific, Waltham, MA, USA). BoDV-1 RNA was detected using two real-time RT-qPCR assays: primers and probe of mix 1 targeting the BoDV-1 X/phosphoprotein (P) gene region and primers and probes of mix 6 targeting the BoDV-1 matrix (M)/glycoprotein (G) gene region [1]. RT-qPCR was performed on a StepOnePlus Real-Time PCR System (Thermo Fisher Scientific). In vitro-transcribed RNA molecules were used as standards for quantification, generated as previously described for a different detection assay [13]. For extraction and inhibition control, samples were spiked with a defined amount of MS2 bacteriophages and a MS2 phage RT-qPCR was performed [14]. All samples were tested in two replicates.

### Next-generation sequencing (NGS)

10 µm-thick sections were cut from a formalin-fixed paraffin-embedded (FFPE) brain tissue block and underwent deparaffinization (Deparaffinization Solution, Qiagen, Hilden, Germany), followed by RNA extraction using the RecoverAll™ Total Nucleic Acid Isolation Kit (Life Technologies, Carlsbad, CA, USA), according to the manufacturer´s instructions. Quality and integrity of the extracted RNA were confirmed using the Qubit™ RNA HS Assay Kit (Thermo Fisher Scientific) and the Agilent 2100 Bioanalyzer (Agilent, Santa Clara, CA, USA). A total amount of 50 ng extracted RNA was subjected to viral metagenomic sequencing on a NextSeq 2000 platform using the 200-cycle (2 × 100 bp paired-end) NextSeq 2000 P2 Reagent Kit (Illumina, San Diego, CA, USA). The raw data generated were cleaned from low-quality reads and polyclonal sequences were removed. The viral genome was obtained using Geneious Prime (Biomatters, Auckland, New Zealand). The nucleotide sequence of the BoDV-1 genome generated in this study has been deposited in GenBank (accession no. PP272805).

### BoDV-1 indirect immunofluorescence assay (iIFA)

A BoDV-1 indirect immunofluorescence assay (iIFA) was performed as previously described [1, 15]. Vero cells persistently infected with BoDV-1 (isolate Regensburg 2020) served for the detection of BoDV-1-reactive IgG antibodies. A secondary Cy-3-conjugated polyclonal rabbit anti-human-IgG antibody (Jackson ImmunoResearch, West Grove, PA, USA) was used in a dilution of 1:200.

### BoDV-1 IgM ELISA system

A BoDV-1 IgM ELISA system was performed as previously described elsewhere [9]. Nunc MaxiSorp plates (Thermo Fisher Scientific) were coated with either recombinant BoDV-1 N, X or P protein. For all samples, a pre-incubation with RF Absorbent (Virion/Serion, Würzburg, Germany) was performed according the manufacturer´s protocol; all samples (serum, CSF) were used in a dilution of 1:100. A secondary polyclonal rabbit anti-human IgM/HRP antibody (Agilent) was used in a dilution of 1:3,000. To control for inter-assay variability, optical density (OD) values were normalised to pre-characterised positive control samples of patients with confirmed BoDV-1 infection.

### BoDV-1 ELISpot

The BoDV-1 ELISpot was performed exactly as previously described elsewhere [12]. Peripheral blood mononuclear cells (PBMC) were isolated from lithium-heparin blood (2 × 7.5 mL) within 24 h after the sample was obtained (pre-analytical storage at room temperature). BoDV-1-derived 11-aa-overlapping 15-mer peptide pools spanning full-length viral nucleoprotein (N), accessory X protein or phosphoprotein (P) (peptides and elephants, Hennigsdorf, Germany) were used to stimulate BoDV-1-specific T cells within the fraction of PBMC. Stimulation with phytohemagglutinin (PHA) solution (T-SPOT.TB Kit, Oxford Immunotec, Abingdon, UK) served as positive control.

### Ethics

The retrospective examination of clinical samples of patients with encephalitis for the detection of new viruses such as BoDV-1 was approved by the Ethics Committee of the Faculty for Medicine, University of Regensburg, Regensburg, Germany (Reference Number: 18–1248-101).

### Graphs

Graphs were created using GraphPad Prism version 10.1.0 (GraphPad Software, San Diego, CA, USA).

## Case presentation

**Fig. 1:**
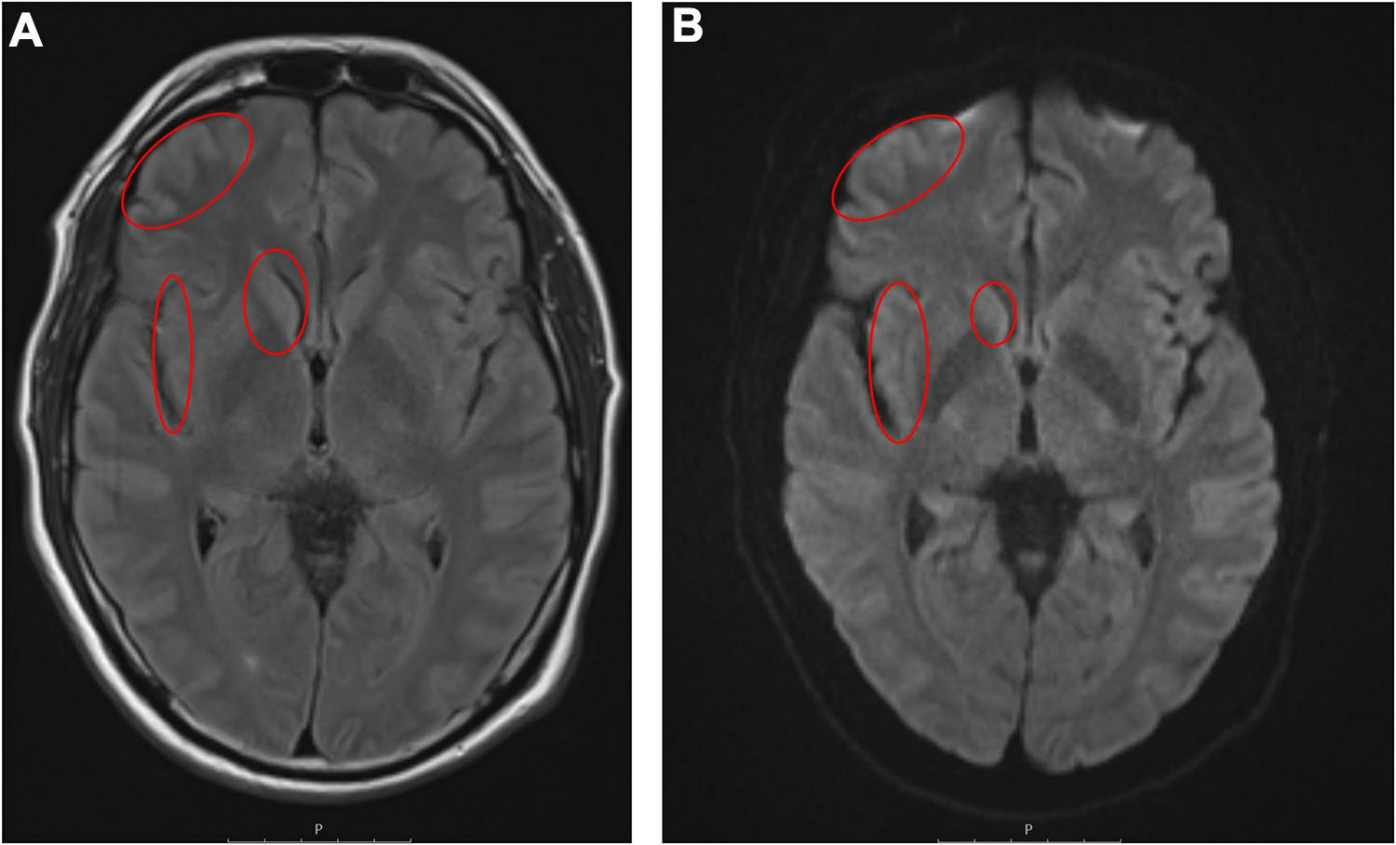
Cerebral MRI obtained on day seven after hospitalisation. The axial fluid-attenuated inversion recovery (FLAIR) sequence shows symmetric elevation in the caudate nuclei, the insular ribbon and the cortex **A**. No contrast media enhancement was visible. An axial diffusion-weighted imaging (DWI) sequence with a corresponding signal elevation (without signal attenuation in apparent diffusion coefficient (ADC) mapping, not shown) is depicted in **B**. For better clarity of the image, symmetric signal elevations were only marked on one side

The 40 − 50-year-old patient (exact age and gender not revealed due to ethical reasons) with no significant past-medical history was admitted to the neurology department of a local hospital within a known endemic region in southern Germany presenting with psychomotor slowing, disorientation and reduced consciousness. About 2.5 weeks prior to admission, the patient had experienced a flu-like illness with fever and headaches and subsequently developed depressive symptoms with exhaustion and reduced psychomotor drive (time course see Table 1). Analysis of a first CSF sample (obtained within three hours on the day of admission) showed mild pleocytosis (14 leukocytes/µL, reference < 5/µL; 93% lymphocytes) and no blood–brain barrier disruption. Acyclovir (750 mg 3x/day) and ceftriaxone (2 g 1x/day) were started as calculated broad-acting anti-infective medication. Ampicillin (2 g 6x/day) and ciprofloxacin (400 mg 2x/day) were added as no clinical improvement was seen. The diagnostic assessment for various infectious agents (multiplex panel: *Escherichia coli, Haemophilus influenzae, Listeria monocytogenes, Neisseria meningitidis, Streptococcus agalactiae, Streptococcus pneumoniae, Cryptococcus neoformans/gattii,* cytomegalovirus, enterovirus, herpes simplex virus 1 and 2, varicella zoster virus, human herpes virus 6, human parechovirus; serology for *Borrelia* and tick-borne encephalitis virus) remained negative. A cMRI performed on day one after admission showed no abnormalities. With suspected autoimmune limbic encephalitis, the patient received high-dose steroids (1000 mg prednisolone for two days, followed by 500 mg prednisolone for further four days), starting at day one after admission, and immune adsorption on day four and five after admission. The diagnostic parameters for autoimmune encephalitis, however, were negative (CASPR2, LGI 1, DPPX, mGLuR5, GABA-A, glycin, CV2/CRMP5, NMDAR). After epileptic seizures occurred, the patient received levetiracetam (1 g 3x/day) and lacosamide (200 mg 2x/day). Despite treatment, the patient´s condition deteriorated and mechanical ventilation was required three days after admission. In a second CSF sample (collected four days after admission, before the start of immune adsorption), white blood count (WBC) (9/µL, reference < 5/µL), protein (520 mg/L, reference < 450 mg/L) and CSF lactate levels (3.2 mmol/L, reference 1.2 − 2.1 mmol/L) were only mildly elevated. An advanced work-up for additional infectious agents (negative PCR for cytomegalovirus, Epstein–Barr virus, enteroviruses, JC virus, measles virus, West Nile virus, Zika virus) was initiated from the second CSF sample. BoDV-1 RNA was positive with 890 copies/mL in CSF, whilst neither IgM antibodies (using a BoDV-1 IgM ELISA system) nor IgG antibodies (using a BoDV-1 iIFA; titre < 10) were detected in the same CSF sample. The examination of two serum samples taken four days (obtained before the start of immune adsorption) and seven days after hospital admission (obtained after immune adsorption on day four and five after hospital admission) also showed negative results for anti-bornavirus IgM and anti-bornavirus IgG. Anti-bornavirus IgG tests in the two sera were performed in two independent laboratories, including the German Consiliary Laboratory for Bornaviruses, located at the Bernhard Nocht Institute for Tropical Medicine, Hamburg, Germany. On day six after admission, the patient remained without sedatives in deep coma and was transferred to a tertiary referral hospital. One day later, a cMRI (Fig. 1) showed extended non-gadolinium-enhancing lesions in the cerebral cortex and medial thalami on both sides. As the disease was at an advanced stage, no further experimental off-label treatment approach was followed and best supportive care was initiated. In a blood sample obtained on day ten after initial hospital admission, IgG seroconversion was detected (iIFA titre 320), whilst no IgM was found using the IgM ELISA system. The patient died on day ten after admission.

**Table 1:** Time course

Timeline	Clinical symptoms	Laboratory tests	Imaging	Therapy	BoDV-1 diagnostics
2.5 weeks prior to hospitalisation	Flu-like illness with fever and headache Followed by depressive symptoms with exhaustion and reduced psychomotor drive				
Day of hospitalisation	Psychomotor slowing Disorientation Reduced consciousness	*CSF:* Leukocytes: 14/µL (reference < 5/µL), 93% lymphocytes No blood–brain barrier disruption Multiplex panel infectious agents: negative (*Escherichia coli, Haemophilus influenzae, Listeria monocytogenes, Neisseria meningitidis, Streptococcus agalactiae, Streptococcus pneumoniae, Cryptococcus neoformans/gattii,* cytomegalovirus, enterovirus, herpes simplex virus 1 and 2, varicella zoster virus, human herpes virus 6, human parechovirus) *CSF/serum:* serology negative for *Borrelia* and tick-borne encephalitis virus		Acyclovir (750 mg 3x/day) Ceftriaxone (2 g 1x/day)	
1 day after hospitalisation			*cMRI: *unremarkable	High-dose steroids (1000 mg prednisolone for 2 days, followed by 500 mg prednisolone for 4 more days)	
3 days after hospitalisation	Clinical deterioration Seizures Intubation			Ampicillin (2 g 6x/day) Levetiracetam (1 g 3x/day)	
4 days after hospitalisation		*CSF:* Leukocytes: 9/µL (reference < 5/µL) Protein: 520 mg/L (reference < 450 mg/L) Lactate: 3.2 mmol/L (reference 1.2 − 2.1 mmol/L) Autoimmune encephalitis tests: negative PCR negative for: cytomegalovirus, Epstein–Barr virus, enteroviruses, JC virus, measles virus, West Nile virus, Zika virus		immune adsorption (day 4 and day 5)	*Serum:* iIFA (IgG): negative (< 20) *CSF:* iIFA (IgG): negative (< 10) RT-qPCR: BoDV-1 RNA 890 copies/mL
5 days after hospitalisation				Ciprofloxacin (400 mg 2x/day) Lacosamide (200 mg 2x/day)	
7 days after hospitalisation			*cMRI:* extended non-gadolinium-enhancing lesions in the cerebral cortex and medial thalami on both sides		*Serum:* iIFA (IgG): negative (< 20) *BoDV-1 ELISpot:* positive
10 days after hospitalisation	The patient died				*serum: *iIFA (IgG): positive (320)

## Results

**Fig. 2:**
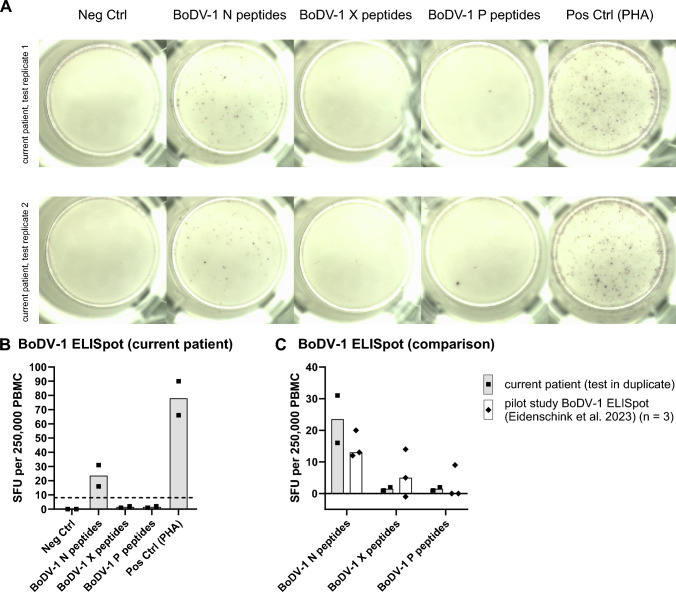
BoDV-1 ELISpot detecting virus-specific T cells on day seven. Peripheral mononuclear cells (PBMC) were isolated from lithium-heparin blood by density gradient centrifugation. A total of 250,000 viable PBMC per well were seeded onto a plate pre-coated with anti-human-IFN-γ (Mabtech). Cells were stimulated with 11-aa-overlapping 15-mer peptide pools spanning full-length BoDV-1 N, X or P protein for 17 − 20 h. Phytohemagglutinin (PHA) served as positive control, medium without additives as negative control. Spots were developed using anti-human-IFN-γ-AP and NBT/BCIP substrate solution (Oxford Immunotec) and analysed by an AID iSpot Robot (AID Autoimmun Diagnostika). The experiment was performed in duplicate from one PBMC sample. **A** shows images of the two replicates as they were taken by the AID iSpot Robot. Spot forming units (SFU) per 250,000 seeded PBMC are shown for the duplicates in **B**. Bars represent the median. The dotted horizontal line gives the cut-off for stimulation with N-derived peptides (> 8 SFU) as it has recently been established (Eidenschink et al. 2023) [12]. In **C**, ELISpot results of the current patient (bars represent median of the duplicate test) are shown in comparison to those of the previously published three patients with BoDV-1 infection (bars represent median of three independent patients) (Eidenschink et al. 2023) [12]

To corroborate the diagnosis, a BoDV-1 ELISpot using fresh lithium-heparin blood obtained seven days after hospital admission was performed for diagnostic purposes. In two replicates from one PBMC sample, median spot forming units (SFU) per 250,000 isolated viable PBMC were 24 (range 16 − 31) for stimulation with peptides spanning the whole viral N protein and 2 (range 1 − 2) for peptides spanning X and P protein, respectively (Fig. 2). As cut-off for the stimulation with N-derived peptides, > 8 SFU/250,000 PBMC had been determined in a previous pilot study [12]. Thus, the ELISpot was interpreted as positive.

From formalin-fixed brain tissue obtained during autopsy, a full-length BoDV-1 genome was obtained at the Consiliary Laboratory by NGS. Phylogenetic analysis assigned this sequence to cluster four, which extends from the Northeast of Bavaria to the federal German states of Thuringia, Saxony-Anhalt and Brandenburg.

## Discussion

BoDV-1 causes rare, but mostly fatal infections in humans. Current seroepidemiological studies did not find evidence for frequent atypical or subclinical courses of infections [15–17]. As the disease is rare, awareness should be further improved, especially in endemic regions within southern and eastern Germany where the occurrence of the virus is well documented in populations of *Crocidura leucodon* and by spill-over infections to sentinel animals (e.g. horses, sheep and alpacas). After an unspecific prodromal phase, psychomotor and neurological symptoms develop. In some cases, neurological symptoms were similar to the presentation of a Guillain–Barré or Miller–Fisher syndrome [1, 15, 18]. The diagnosis of BoDV-1 infections, however, might be challenging. As the virus is cell-associated, only low copy numbers are shed into lumbar CSF. A recent study estimated a sensitivity of 25 − 67% for BoDV-1 RT-qPCR in CSF [17]. Thus, it is recommended to perform serology from a serum sample in parallel. However, the same study reported detection of antibodies at a median of 32 days after hospitalisation (41 days after symptom onset); seroconversion, if documented, was seen on days 3 − 24 after hospitalisation [17]. In our reported case, BoDV-1 RNA was detected in CSF, while no BoDV-1-reactive IgG antibodies were found in CSF or serum four days after hospitalisation before the start of immune adsorption. Whilst antibodies in serum were still negative seven days after hospitalisation, BoDV-1-specific T cells were detected using a recently published BoDV-1 ELISpot protocol [12]. Admittedly, corticosteroid use and the immune adsorption performed on day four and five after hospitalisation may have interfered with the negative follow-up serology on day seven; anti-bornavirus IgG was first detected on day ten with a titre of 320 in iIFA. Regarding the ELISpot test, the virus-specific T cells were directed against the BoDV-1 N protein, which also was described as major target of T cell-mediated immune responses in animal models, as it is expressed earliest and most abundantly, at least in the early infection phase [19]. Whether the undetected T-cell response against other viral proteins such as the X and P protein in the current case is due to the viral protein stoichiometry, viral protein expression kinetics, compartmentalization of virus-specific T cells, or due to immunological factors remains elusive. In previous studies, at least some patients showed reactive T cells against the viral X and P protein (Fig. 2C) [12].

This is the first time that a positive BoDV-1 ELISpot was obtained on day seven at a comparatively early stage during the course of the disease when serology was still negative, either naturally or due to prior immune absorption, whilst in our previous BoDV-1 ELISpot pilot study, PBMC samples from three patients with confirmed BoDV-1 infection were only available from later stages of the disease (second to third week after hospitalisation) [12]. Our result demonstrates that the BoDV-1 ELISpot serves as an additional diagnostic tool even when serology might be still negative or immune adsorption interferes with serological tests.

## Data Availability

Sequence data of the BoDV-1 genome generated in this study have been deposited in GenBank (accession no. PP272805). ELISpot and serological data generated in this report are available from the corresponding author Ma.Ba. on reasonable request.
